# Oculomotor corollary discharge signaling is related to repetitive behavior in children with autism spectrum disorder

**DOI:** 10.1167/jov.21.8.9

**Published:** 2021-08-05

**Authors:** Beier Yao, Martin Rolfs, Christopher McLaughlin, Emily L. Isenstein, Sylvia B. Guillory, Hannah Grosman, Deborah A. Kashy, Jennifer H. Foss-Feig, Katharine N. Thakkar

**Affiliations:** 1 Department of Psychology, Michigan State University, East Lansing, MI, USA; 2 Department of Psychology, Humboldt-Universität zu Berlin, Germany; 3 Seaver Autism Center, Icahn School of Medicine at Mount Sinai Hospital, New York, NY, USA; 4 Department of Brain and Cognitive Sciences, University of Rochester, Rochester, NY, USA; 5 Department of Psychiatry, Icahn School of Medicine at Mount Sinai Hospital, New York, NY, USA; 6 Division of Psychiatry and Behavioral Medicine, Michigan State University, Grand Rapids, MI, USA

**Keywords:** efference copy, sensory hyporesponsiveness, saccadic eye movements, trans-saccadic perception, remapping

## Abstract

Corollary discharge (CD) signals are “copies” of motor signals sent to sensory regions that allow animals to adjust sensory consequences of self-generated actions. Autism spectrum disorder (ASD) is characterized by sensory and motor deficits, which may be underpinned by altered CD signaling. We evaluated oculomotor CD using the blanking task, which measures the influence of saccades on visual perception, in 30 children with ASD and 35 typically developing (TD) children. Participants were instructed to make a saccade to a visual target. Upon saccade initiation, the presaccadic target disappeared and reappeared to the left or right of the original position. Participants indicated the direction of the jump. With intact CD, participants can make accurate perceptual judgements. Otherwise, participants may use saccade landing site as a proxy of the presaccadic target and use it to inform perception. We used multilevel modeling to examine the influence of saccade landing site on trans-saccadic perceptual judgements. We found that, compared with TD participants, children with ASD were more sensitive to target displacement and less reliant on saccade landing site when spatial uncertainty of the post-saccadic target was high. This pattern was driven by ASD participants with less severe restricted and repetitive behaviors. These results suggest a relationship between altered CD signaling and core ASD symptoms.

## Introduction

Despite frequent saccadic eye movements that cause retinal displacement of visual input, we are able to maintain a stable perception of the world—an important function supported by corollary discharge (CD) signals. CD signals are “copies” of motor commands that are sent to sensory areas in the brain. They are ubiquitous across the animal kingdom and support the critical computation of distinguishing sensory input brought about by our own movements from sensory input caused by external forces (Crapse & Sommer, 2008; Sommer & Wurtz, 2008). These CD signals allow sensory brain regions to compute the predicted consequences of the imminent movement and to modulate their response to that input in a manner that enhances processing efficiency. For example, our visual system may use CD signals to predict and therefore account for the imminent changes in visual input caused by saccades (Wurtz, 2018) and plan saccades in parallel, thus enabling rapid sequential eye movements (Becker & Jürgens, 1979). Accordingly, CD signaling may be a foundational building block linking action to perception, and disturbed CD signaling may lead to downstream consequences such as sensory and motor symptoms in autism spectrum disorder (ASD).

ASD is a neurodevelopmental disorder that is diagnosed on the basis of difficulties in social interaction and communication and restricted and repetitive patterns of behavior, which include stereotyped and perseverative movements (e.g., hand flapping), insistence on sameness (e.g., rigidly adhering to the same routine), and fixed interests. Individuals with ASD also experience alterations in sensory processing, including both hyporesponsiveness (e.g., indifference to pain, heat, or cold) and hyper-responsiveness (e.g., disturbed by everyday noises). Accumulating evidence suggests that sensory symptoms are in fact a core phenotype of ASD because they are present in 90% of persons with ASD (Tavassoli, Miller, Schoen, Nielsen, & Baron-Cohen, 2014; Tomchek & Dunn, 2007) and can be observed as early as infancy (Baranek et al., 2013). Therefore, sensory processing abnormality was recently added to the diagnostic criteria of ASD (American Psychiatric Association, 2013). These symptoms may have downstream consequences for more complex behaviors, particularly with respect to restricted and repetitive behaviors.

Indeed, several studies have reported associations between sensory processing abnormalities and restricted and repetitive behaviors in ASD (Boyd, McBee, Holtzclaw, Baranek, & Bodfish, 2009; Foss-Feig, Heacock, & Cascio, 2012; Wigham, Rodgers, South, McConachie, & Freeston, 2015; Wolff et al., 2017). Specifically, more severe repetitive behaviors have been related to greater hyporesponsiveness across sensory modalities (Wigham et al., 2015; Wolff et al., 2017) and tactile hyporesponsiveness in particular (Foss-Feig et al., 2012). These associations do not seem to arise from a common higher-order psychological or cognitive deficit (Boyd et al., 2009; Wigham et al., 2015). Furthermore, sensory symptoms and restricted and repetitive behaviors, but not social–communicative deficits, may share structural brain correlates in the cerebellum and corpus callosum (Wolff et al., 2017). Despite these tantalizing relationships, exactly how sensory and motor symptoms are related remains unclear. We propose that an alteration in the link between action and perception (i.e., altered CD signaling) may underwrite sensory and motor (i.e., restricted and repetitive behaviors) symptoms in ASD.

**Figure 1.**
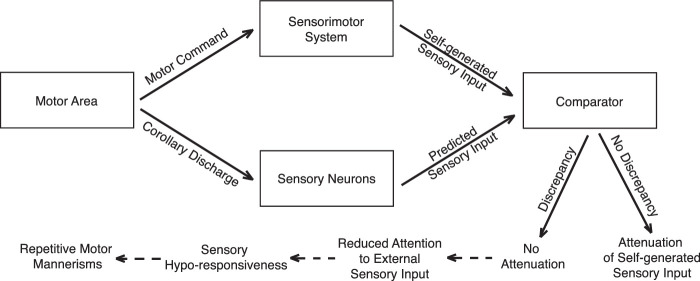
A conceptual model for how altered CD signaling may lead to sensory hyporesponsiveness and repetitive behaviors in ASD. CD signals are “copies” of a motor command that are sent to the sensory neurons in the brain. They can be used to generate a prediction of sensory input caused by the self-generated actions. The predicted sensory input is then compared with the actual sensory input. When there is no discrepancy between the two, the sensory consequences of self-generated actions can be anticipated to increase processing of more relevant external sensory signals. In contrast, a discrepancy may lead to a failure in appropriately modulating the sensory consequences, and thus to an abnormally salient experience of that input. Consequently, this may lead to decreased attention to external sensory signals and sensory hyporesponsiveness. Meanwhile, a lack of stimulation by external sensory input may lead to attempts of generating sensory input through one's own actions, resulting in repetitive motor mannerisms.

CD signals can serve to anticipate sensory consequences of self-generated actions to elevate the processing of more relevant external sensory signals (Bridgeman, Hendry, & Stark, 1975; Crapse & Sommer, 2008; Ford & Mathalon, 2004). When CD signaling is intact, the predicted sensory consequences of self-generated movements are consistent with the actual sensory input, and the resulting sensory experiences can thus be attenuated. If CD signaling goes awry, then sensory neurons will not properly modulate the response to self-generated input (Cullen, 2004; Poulet & Hedwig, 2006; Whitford, Ford, Mathalon, Kubicki, & Shenton, 2012). Given the presence of both sensory and motor symptoms in ASD, we posit that altered CD signaling may have several downstream consequences that are relevant to the ASD phenotype. First, a failure to appropriately modulate the sensory response to self-generated input may result in an abnormally salient experience of that input. This process may in turn lead to decreased attention to external sensory signals, and manifest behaviorally as sensory hyporesponsiveness. At the same time, when a person is understimulated by external sensory input, they may look inward to generate enough sensory input through their own actions, resulting in repetitive motor mannerisms (Ornitz, 1974). Therefore, we hypothesize that abnormal CD signaling may be a candidate mechanism underlying sensory hyporesponsiveness and repetitive behaviors in ASD (Figure 1; see also Foss-Feig et al., 2012).

One way in which CD signals exert their influence in the visuomotor system is via predictive remapping, which describes the property of a subset of visual neurons whereby they begin responding to a visual stimulus before a prepared saccade brings it into their receptive field (Cavanagh, Hunt, Afraz, & Rolfs, 2010; Duhamel, Colby, & Goldberg, 1992; Inaba & Kawano, 2014; Nakamura & Colby, 2002; Umeno & Goldberg, 1997; Walker, Fitzgibbon, & Goldberg, 1995). At the neural level, predictive remapping allows visual brain areas to prepare for the imminent displacement of the retinal image. CD signals convey the information about the impending saccade kinematics that are crucial for predictive updating of neuronal activity (Mirpour & Bisley, 2012; Wurtz, 2008). Findings from primate neurophysiology and human lesion studies have highlighted a circuit whereby CD signals originating in brainstem and midbrain saccade generation neurons are routed to cortical visual and visuomotor neurons via the thalamus and cerebellum (Stone & Lisberger, 1990).

**Figure 2.**
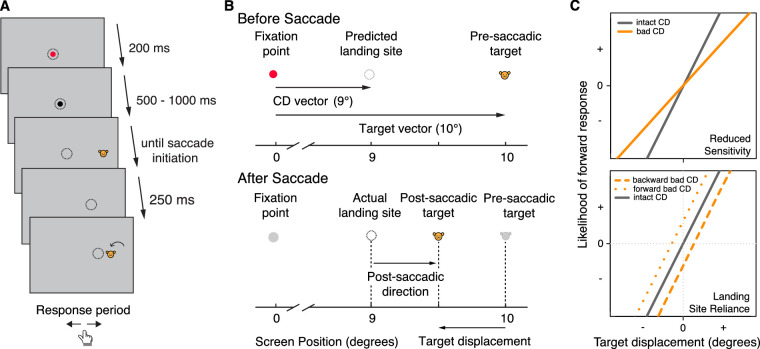
(A) An example trial of the blanking task. Dotted circles indicate gaze positions. The arrow on the last screen indicates the direction of target displacement. Dotted circles and the arrow do not appear in the actual task. (B) Making perceptual judgments on a trial. *Top*, Once the stimulus appears at the presaccadic target location, a motor command is generated to execute a saccade, and a CD vector associated with the command is computed at the same time. On this example trial, the predicted saccade landing site (based on an accurate CD signal) will fall short of the target. *Bottom*, Upon saccade initiation, the stimulus will disappear and reappear at the post-saccadic target location, which can be to the left or right of the presaccadic target location. If the participant can use the CD signal to remap the location of the presaccadic target, then they should be able to answer according to the actual target displacement. On this example trial, the post-saccadic target location is to the left of the presaccadic target location, so the participant should judge it as a backward displacement. However, if the participant has altered CD signaling, they may use saccade landing site as a proxy of the presaccadic target location. In this case, the participant will answer according to the post-saccadic direction (i.e., the post-saccadic target location was forward to where their eye landed) and indicate a forward displacement instead. Adapted from Collins et al. (2009). (C) Illustrations of hypothesized data. The vertical axis represents the likelihood of making a forward response (positive numbers indicate that participants are more likely to answer forward; negative numbers indicate more likely to answer backward). *Top*, A lesser influence of CD signals (or an unaltered influence of inaccurate or imprecise CD signals) would be expected to result in a decreased sensitivity to target displacement (i.e., a flatter slope). *Bottom*, A lesser influence of CD signals may lead to participants relying on saccade landing site as a proxy of the presaccadic target location. This tendency will manifest as a positive intercept (i.e., a forward response bias) when the post-saccadic target location was forward to their saccade landing site, and/or a negative intercept (i.e., a backward response bias) when the post-saccadic target location was backward to their saccade landing site. Note that an inaccurate or imprecise CD signal alone—with no reliance on saccade landing site—would also predict a flatter slope (top), but it would not predict a change in intercept as a function of saccade landing site (bottom).

Predictive remapping can be measured behaviorally using the blanking task (Deubel, Schneider, & Bridgeman, 1996). In this task, a saccade target is presented, prompting the participant's saccade, and then extinguished upon saccade onset (Figure 2). After a brief delay, the target reappears near its initial (presaccadic) location. The participant must then indicate the perceived direction of the displacement. The key question here is: what information will participants use to inform this perceptual judgement? Saccades are often not accurate, falling long or, more often, short of the target. Accurate localization of the new (i.e., post-saccadic) target location relies on an accurate CD signal that conveys the kinematics of the actual (rather than ideal) saccade vector. The perceptual system can then use this CD signal to remap the presaccadic target location and thus correctly localize the presaccadic target. Dysfunction in CD signaling, however, would result in a failure to remap the presaccadic target location and may lead observers to rely on the post-saccadic eye position as a proxy for the presaccadic target location. Consistent with having an intact CD signal, healthy observers do not show a reliance on post-saccadic eye position in this task (Collins, Rolfs, Deubel, & Cavanagh, 2009); however, non-human primates with reversible inactivation of the mediodorsal nucleus of the thalamus and humans with lesions to the same region do (Cavanaugh, Berman, Joiner, & Wurtz, 2016; Ostendorf, Liebermann, & Ploner, 2010), consistent with the role of this region in relaying oculomotor CD signals. A similar reliance on post-saccadic eye position has also been observed in more symptomatic participants with schizophrenia (Bansal, Bray, Schwartz, & Joiner, 2018; Rösler et al., 2015). Schizophrenia, like autism, is considered a neurodevelopmental disorder with core sensory and motor abnormalities (Insel, 2010).

The current study aims to investigate CD signaling by testing trans-saccadic perception using the blanking task in a group of typically developing (TD) children and children with ASD and to explore putative relationships between indices of the oculomotor mechanisms supporting trans-saccadic perception and the severity of clinical sensory and motor features of ASD. We hypothesized that putative alterations in CD signaling would lead to an increased reliance on saccade landing site in the ASD group relative to controls when judging the direction of target displacement. Moreover, given the role of CD signals in suppressing sensory consequences of self-generated actions, we further hypothesized that the severity of sensory hyporesponsiveness and repetitive motor behaviors would be associated with a reliance on the saccade landing site in the ASD group. Results from this study will add to our understanding of connections between motor and sensory processing symptoms in ASD, as well as address the putative role of CD signaling as a common underlying mechanism of these symptoms.

## Methods

### Participants

Thirty-six children and adolescents with ASD and 37 TD controls completed the blanking task. After examining task performance, eight participants (six ASD, two TD) were excluded (exclusion criteria detailed in the Data Analysis section), resulting in a final sample of 30 ASD and 35 TD participants (see Table 1 for demographic and clinical data). A diagnosis of ASD based on criteria from the *Diagnostic* *and* *Statistical* *Manual of Mental Disorders*, fifth edition, was established by research-reliable, licensed clinical psychologists using the Autism Diagnostic Observation Schedule (Lord et al., 2012), Autism Diagnostic Interview—Revised (ADI-R) (Rutter, Le Couteur, & Lord, 2003) and clinical judgment. Participants in the TD group were excluded if they had a history of any psychiatric or neurodevelopmental disorder or a first-degree relative with idiopathic ASD. Participants in both groups were excluded if they had a history of neurological disorder (including seizures and head trauma), did not have normal or corrected-to-normal hearing and vision, or had IQ of less than 70. IQ was assessed with the Wechsler Adult Intelligence Scale (Wechsler, 2008), Wechsler Intelligence Scale for Children (Wechsler, 2014), or the Wechsler Abbreviated Scale of Intelligence (Wechsler, 2011). The current experiment was administered as part of a larger battery that included many clinical measures and other behavioral and psychophysiological tasks. The clinical measures of interest to this study included the ADI-R, Repetitive Behavior Scale–Revised (RBS-R), and the Sensory Experience Questionnaire 3.0 (SEQ). The repetitive behaviors domain score from the ADI-R was used to capture past and current repetitive behavior in participants with ASD. The RBS-R was administered to the parents to measure the breadth of current repetitive behavior in participants (Lam & Aman, 2007). The SEQ was administered to the parents to measure participants’ behavioral responses to common everyday sensory experiences (Baranek, David, Poe, Stone, & Watson, 2006). For this study, we only used the sensory hyporesponsiveness subscale from the SEQ. TD participants and participants with ASD did not differ significantly on age, sex, or education. The TD group had significantly higher average IQ than the ASD group.

**Table 1.** Demographic and clinical information.

	TD (*n* = 35)	ASD (*n* = 30)		
	Mean ± SD	Mean ± SD	Statistics	*p* Value
Age (years)	12.86 ± 2.62	12.46 ± 2.72	*t* = −0.61	0.55
Sex (M/F)	22/13	22/8	*χ^2^*(1) = 0.81	0.43
IQ^a^	111.68 ± 18.62	101.67 ± 19.27	*t* = −2.11	0.04
Race				
American Indian and Alaska Native	0	1	*χ^2^*(5) = 6.10	0.30
Asian	1	0		
Black	12	5		
Multiracial	7	10		
White	12	13		
Unknown	3	1		
Hispanic/Latino (Y/N)	10/23	3/27	*χ^2^*(1) = 3.96	0.06
Household income^b^	2.59 ± 2.31	3.83 ± 2.21	*χ^2^*(6) = 13.54	0.04
ADOS Algorithm Domain and Summary Scores^c^				
Social affect total score	–	13.27 ± 4.19	*–*	–
Restricted and repetitive behaviors	–	3.33 ± 1.77	*–*	–
ADI-R Algorithm Domain Scores^c^				
Language/communication	–	16.50 ± 4.49	*–*	–
Reciprocal social interactions	–	19.73 ± 4.65	*–*	–
Restricted and repetitive behaviors	–	6.70 ± 1.75	*–*	–
RBS-R Total Score^c^	–	19.93 ± 15.06	*–*	–
SEQ: Hyper-responsiveness subscale total score^c^	–	39.44 ± 21.86	–	–
SEQ: Hyporesponsiveness subscale total score^c^	–	14.98 ± 9.48	*–*	–
Nonsocial items total score	–	9.43 ± 6.57	*–*	–
Social items total score	–	5.55 ± 3.70	–	–

Notes: ASD, children with autism spectrum disorder; TD, typically developing children.

a Based on the Wechsler Adult Intelligence Scale (WAIS-IV), Wechsler Intelligence Scale for Children (WISC-V), and the Wechsler Abbreviated Scale of Intelligence (WASI-II).

b Household income category (annual): 0 = $0–$24,999, 1 = $25,000–$49,000, 2 = $50,000–$74,999, 3 = $75,000–$99,999, 4 = $100,000–$149,999, 5 = $150,000–$199,999, 6 = $200,000+.

c The possible range of scores for each symptom measure is as follows: Autism Diagnostic Observation Schedule (ADOS) social affect 0–20, ADOS restricted and repetitive behaviors 0–10, ADI-R language/communication 0–26, ADI-R reciprocal social interactions 0–30, ADI-R restricted and repetitive behaviors 0–12, RBS-R 0–129, SEQ hyper-responsiveness 0–124, SEQ hyporesponsiveness 0–72 (social items 0–16 and nonsocial items 0–56).

All participants’ legal guardians gave written informed consent and all participants gave informed assent. Participants were compensated for participation. The study was approved by the Mount Sinai Program for the Protection of Human Subjects.

### Blanking task

#### Apparatus and stimuli

Participants sat in a dimly lit room in front of a computer screen (screen size: 338 × 269 cm; spatial resolution: 1280 × 1024 pixels; refresh rate: 60 Hz; distance to screen: 60 cm). The initial fixation point was a red dot (that later turned black) with a diameter of 0.2° presented on a grey background. The target stimulus was the head of a cartoon tiger with a diameter of 0.5°. Eye position was tracked by an EyeLink 1000 (SR Research, Ottawa, Ontario, Canada). Responses were recorded by a computer keyboard. MATLAB (MathWorks, Portola Valley, CA) was used to present the stimulus and collect responses through the Psychophysics (Brainard, 1997) and EyeLink (Cornelissen, Peters, & Palmer, 2002) toolboxes.

#### Design and procedure

The blanking task putatively indexes the degree to which visual perception is affected immediately after a saccade. Participants started each trial by fixating on a red dot (Figure 2A). The dot randomly appeared at one of three locations (a −1°, 0°, or 1° displacement horizontally relative to the center of the screen) with equal probability to reduce anticipation effects or stereotypical behavior (Collins et al., 2009). Once fixation was maintained for 200 ms, the dot turned black, signaling that the target would appear shortly. After a random delay of 500 to 1000 ms, the dot disappeared and a cartoon character (target) appeared at a new location 10° to the left or right of the fixation position (presaccadic location). The participant was told to look at the target as soon as possible. Once a saccade was detected, the target would disappear (i.e., blank) for 250 ms and reappear at a location (post-saccadic location) that was displaced horizontally relative to the presaccadic location. The target was displaced by 3.00°, 2.00°, 1.50°, 1.00°, 0.50°, or 0.25° to the right or left of the presaccadic target or appeared in the same location as the presaccadic target (i.e., 0° displacement). The target then stayed on the screen until a response was detected. The participant then indicated via a key press in which direction the target jumped relative to its presaccadic location (i.e., left or right). For analysis purposes, we recoded these responses as follows: “forward” refers to target jumping away from the initial fixation position; and “backward” refers to target jumping toward the initial fixation. Thus, the key outcome is a dichotomous variable denoting whether the participant judged the target jumping forward or backward. The combination of three fixation positions × 13 post-saccadic displacements × two saccade directions (left, right) × three runs resulted in 234 total trials. We used a boundary technique to perform online saccade detection: saccade initiation was operationalized as the detection of eyes leaving a 2° window around the initial fixation dot. The same 2° window was also used to ensure stable fixation at the start of the trial and before the appearance of the presaccadic target (i.e., during the random delay after the fixation dot turned black). Participants would receive a warning message if they initiated a saccade before the target onset, and the trial would be repeated.

### Data analysis

Saccades were detected offline using the automated EyeLink procedure (velocity of >30°/s, acceleration of >8,000°/s^2^, and displacement of >0.1°). Response saccades were defined as the first saccade initiated at least 100 ms after presaccadic target presentation, larger than 1°, and landed within 8° of the presaccadic target location. Trials were excluded if no valid response saccades were identified.

Participants were excluded based on two indices of task performance. First, we computed overall response accuracy using the signal detection theory (Green & Swets, 1966). Specifically, we defined the hit rate as the proportion of forward responses out of all forward displacement trials and the false alarm rate as the proportion of forward responses out of all backward displacement trials (trials with a 0 displacement were excluded from this calculation). We then calculated the sensitivity index (*d’*) as the *z*-score of the hit rate minus the *z*-score of the false alarm rate. Participants with a *d’* of less than 1 were excluded from all analyses. Next, we computed the percentage of forward responses as a function of target displacement, collapsing across initial fixation positions and saccade directions. A four-parameter logistic function was fit to each participant's data:

$$f\left(x\right)=a+\frac{\left(d-a\right)}{1+10^{b(c-x)}}$$

Where *a* is the minimum value, *d* is the maximum value, *b* is the slope, and *c* is the point midway between *a* and *d*. Using this function, we determined the perceptual null location (PNL) for each participant—the displacement where participants perceived no difference between the presaccadic and post-saccadic target locations. In other words, the PNL is the post-saccadic target location where the proportion of forward and backward responses were equal (i.e., the 50% point on the logistic function). Participants with an absolute value of PNL more than 2 were excluded from all analyses. Using the *d’* and PNL exclusion criteria, we excluded two TD children and six participants with ASD in total. Note that the logistic function was only used to identify and exclude participants who did not perform the task properly and was not part of our main statistical analyses. The data files and analysis scripts can be accessed via the following link: https://osf.io/q7r2n/.

### Statistical analysis

Independent *t* tests were used to compare the TD and ASD groups on age, IQ, *d'*, PNL, mean saccade amplitude, mean reaction time to initiate the first saccade, mean variability in saccade end point (quantified as the standard deviation [SD] of the absolute distance between the initial saccade end point and the presaccadic target location), and percentage of invalid trials. We conducted χ^2^ tests to compare groups on sex, education, race, ethnicity, and annual household income. These statistical analyses were conducted using SPSS Statistics version 25.0 (IBM, Armonk, NY).

We used multilevel modeling using a binary logistic model with maximum pseudo-likelihood to predict participants’ perceptual judgment on each trial (i.e., forward vs. backward). Because the position of the initial fixation circle did not significantly predict participants’ responses, it was not included as a predictor in any of the models. For analysis purposes, we defined two variables quantifying saccade landing site information on each trial: post-saccadic direction and post-saccadic distance (Figure 2B). The post-saccadic direction refers to whether the post-saccadic target location was forward to the saccade landing site (i.e., saccade landing site was in between fixation and post-saccadic location) or backward (i.e., post-saccadic location was in between fixation and saccade landing site). If participants used saccade landing site as a proxy for the presaccadic location, we would expect to see a high likelihood of forward response on forward appearing trials and backward response on backward appearing trials. Post-saccadic distance refers to the absolute value of the distance between saccade landing site and post-saccadic location of the target: the greater the distance, the greater the spatial uncertainty of the post-saccadic target (owing to the post-saccadic target being farther from the fovea). In principle, this spatial uncertainty should be independent of CD functioning. However, because participants need to compare the post-saccadic target location to the remapped presaccadic location (supported by CD signaling), the uncertainty of post-saccadic target location from saccade landing site could influence the difficulty of perceptual judgments, with perceptual judgments being more difficult on trials where the post-saccadic target appeared further away from the saccade landing site.

Multilevel modeling was conducted using SAS version 9.4 (SAS Institute, Cary, NC). In our first model, we tested the effect of group along with several other task factors, as described elsewhere in this article. In a second set of models, we examined whether task performance varied as a function of clinical symptom severity in participants with ASD only. Because parameter estimates in log odds are difficult to interpret, we calculated the corresponding odds by taking the exponent of log odds and reported odds for all analysis results.

In the first analysis model, task factors included post-saccadic direction and post-saccadic distance to examine whether participants relied on their saccade landing site as a proxy for presaccadic target location (instead of using CD information). In addition, we included target displacement. Participant response was coded as 1 = forward or 0 = backward. Target displacement was coded such that a positive number refers to a forward displacement (e.g., an 0.5° displacement means the target jumped 0.5° away from the initial fixation position) and a negative number refers to a backward displacement (e.g., a −3° displacement means the target jumped 3° toward the initial fixation). Therefore, the proportion of forward responses is expected to increase as a function of displacement. Finally, we included saccade direction to examine potential laterality effects. Saccade direction was effect coded as −1 = leftward or 1 = rightward, the group was effect coded as −1 = TD children or 1 = children with ASD, and the post-saccadic direction was effect coded as −1 = forward or 1 = backward. Post-saccadic distance was grand mean centered. All main effects and interactions among these five variables (group, saccade direction, target displacement, post-saccadic direction, and post-saccadic distance) were included in the first model. When there were significant interactions, we computed simple slopes for separate conditions for follow-up analyses (Tabachnick & Fidell, 2013). In other words, we probed the effect of predictor A on the response variable within different levels of predictor B, by deriving simple regression slopes for selected values of B. When B was a categorical variable, we derived simple slopes for each category (e.g., children with ASD vs. TD children). When B was a continuous variable, we derived simple slopes for high (i.e., 1 SD above the mean) and low (i.e., 1 SD below the mean) values of B.

When interpreting the multilevel model results, we focused on two key parameters: the slope of the target displacement and the intercept. The slope indexes participants’ sensitivity to target displacement and is conceptually equivalent to the just noticeable difference parameter in the conventional psychometric curve described elsewhere in this article. In other words, greater perceptual sensitivity is indexed by a steeper slope of the relationship between perceptual judgments and target displacement. The intercept captures participants’ response bias when the actual target displacement is 0, and thus can be seen as conceptually equivalent to the PNL parameter in the conventional psychometric curve described above. Here, a positive intercept would suggest a bias toward responding “forward” and a negative intercept would suggest a bias toward responding “backward.”

We expected that an increase in target displacement would predict an increase in the likelihood of a forward response (i.e., the larger the forward jump, the higher the likelihood of reporting forward). Further, we predicted that the influence of CD would be decreased in participants with ASD, leading them to rely less on the actual target displacement and more on the saccade landing site; this property should manifest in an attenuated effect of target displacement on forward judgements (i.e., a flatter slope) in participants with ASD (Figure 2C, top). We did not expect post-saccadic direction to be a significant predictor of perceptual judgment in participants with intact CD signals. However, if participants with ASD have a decreased influence of CD signals, as hypothesized, and thus use the saccade landing site as a proxy of the presaccadic target location, we expected an increased likelihood of making a forward response on trials where the post-saccadic target appeared forward of their gaze location, and a decreased likelihood of forward responses on backward trials (i.e., a non-0 intercept; Figure 2C, bottom). We did not have specific hypotheses regarding the effect of post-saccadic distance on perceptual judgment. To account for individual differences in performance generally, as well as individual differences in the effects of the task factors, random effects included variances for the intercepts, as well as variances of the slopes for target displacement, saccade direction, post-saccadic direction, post-saccadic distance, target displacement × saccade direction, saccade direction × post-saccadic direction, saccade direction × post-saccadic distance, target displacement × post-saccadic direction, and post-saccadic direction × post-saccadic distance. In sum, the first model can be expressed in the following regression equations, where *i* stands for the trial, and *j* stands for the participant:

Lower level (i.e., trial-level) equation:

$$\begin{array}{ccc} Response_{ij} & = & \beta_{0j}+\beta_{1j}\left(Saccade\;Direction_{ij}\right)\\ & & +\beta_{2j}\left(Target\;Displacement_{ij}\right)\\ & & +\beta_{3j}\left(Post-saccadic\;Direction_{ij}\right)\\ & & +\beta_{4j}\left(Post-saccadic\;Distance_{ij}\right)\\ & & +\beta_{5j}(Saccade\;Direction_{ij}\\ & & \times Target\;Displacement_{ij})\\ & & +\beta_{6j}(Saccade\;Direction_{ij}\\ & & \times Post-saccadic\;Direction_{ij})\\ & & +\beta_{7j}(Saccade\;Direction_{ij}\\ & & \times Post-saccadic\;Distance_{ij})\\ & & +\beta_{8j}(Target\;Displacement_{ij}\\ & & \times Post-saccadic\;Direction_{ij})\\ & & +\beta_{9j}(Post-saccadic\;Direction_{ij}\\ & & \times Post-saccadic\;Distance_{ij})\\ & & +\beta_{10j}(Target\;Displacement_{ij}\\ & & \times Post-saccadic\;Distance_{ij})\\ & & +\beta_{11j}(Saccade\;Direction_{ij}\\ & & \times Target\;Displacement_{ij}\\ & & \times Post-saccadic\;Direction_{ij})\\ & & +\beta_{12j}(Saccade\;Direction_{ij}\\ & & \times Target\;Displacement_{ij}\\ & & \times Post-saccadic\;Distance_{ij})\\ & & +\beta_{13j}(Saccade\;Direction_{ij}\\ & & \times Post-saccadic\;Direction_{ij}\\ & & \times Post-saccadic\;Distance_{ij})\\ & & +\beta_{14j}(Target\;Displacement_{ij}\\ & & \times Post-saccadic\;Direction_{ij}\\ & & \times Post-saccadic\;Distance_{ij})\\ & & +\beta_{15j}(Saccade\;Direction_{ij}\\ & & \times Target\;Displacement_{ij}\\ & & \times Post-saccadic\;Direction_{ij}\\ & & \times Post-saccadic\;Distance_{ij}) \end{array}$$

Upper level (i.e., participant-level) equations:

For *k* = 0–9:

$$\beta_{kj}=\gamma_{k0}+\gamma_{k1}\left(Group_{j}\right)+u_{kj}$$

For *k* = 10 – 15:

$$\beta_{kj}=\gamma_{k0}+\gamma_{k1}\left(Group_{j}\right)$$

We next examined whether task performance varied as a function of clinical symptom severity in participants with ASD only. We examined the effects of three measures of ASD symptoms, including the repetitive behaviors domain score from the ADI-R, the RBS-R total score, and the hyporesponsiveness subscale total score from the SEQ. In each of these models, the clinical measure was included as a main effect and as a moderator of the effects of the task factors. We hypothesized that participants with ASD with more severe sensory and motor clinical symptoms would exhibit response patterns indicative of a decreased influence of CD on visual perception (e.g., attenuated sensitivity to target displacement, a reliance on saccade landing site). Because saccade direction showed no significant effects in the first model, this factor was removed from the fixed effects structure of the model in these secondary analyses to reduce model complexity. Saccade direction was, nonetheless, preserved in the random effects structure owing to significant intersubject variability in the effect of saccade direction on task performance. Therefore, random effects included variances for the intercepts, as well as variances of the slopes for target displacement, saccade direction, post-saccadic direction, target displacement × saccade direction, saccade direction × post-saccadic distance, and post-saccadic direction × post-saccadic distance. Two participants did not complete the RBS-R questionnaire and were therefore not included in the RBS-R model. One participant did not have data on the SEQ questionnaire and was therefore not included in that model. Another participant missed two of the 18 items constituting the hyporesponsiveness subscale. No particular pattern was detected in missing data, so this participant's response was deemed valid and mean imputation was used to calculate a subscale total score. Distributions of all three clinical measures were examined and no statistical outliers were observed. All clinical measures were grand mean centered.

Finally, in an exploratory analysis, we examined the metrics of corrective saccades to the post-saccadic target and their effect on task performance. Methods and results are detailed in Supplementary Material. In short, corrective saccade metrics did not differ between groups, and including corrective saccade execution in our models did not meaningfully change the results. We also examined whether there was a moderation effect of IQ on task performance and on the relationship between repetitive behaviors and task performance. There were no significant main or interaction effects involving IQ (see Supplementary Material).

## Results

### Saccade metrics and task performance

**Figure 3.**
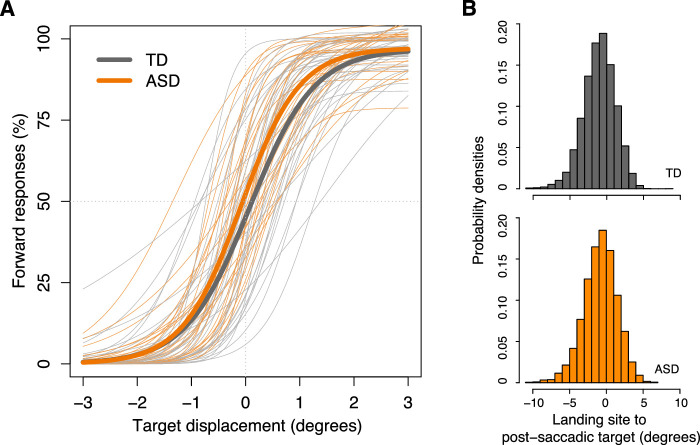
(A) Four-parameter (minimum value, maximum value, midway point between the minimum and maximum values, and slope) logistic fits of percentage of forward responses as a function of target displacement. Thicker lines represent fits of the group averages and thinner lines represent individual participants’ fits. Note that this graph is for visualization purpose only and does not reflect the main multilevel analyses. (B) Probability density graphs of the distance from saccade landing site to post-saccadic target locations, separated by group. On the *x*-axis, negative values indicate that relative to the post-saccadic target location, the landing site was closer to the initial fixation point. Positive values indicate that relative to the post-saccadic target location, the landing site was further away from the fixation point. ASD, children with autism spectrum disorder; TD, typically developing children.

The two groups did not differ significantly on *d'*, PNL, mean saccade amplitude, mean reaction time to initiate the first saccade, or mean variability in saccade end point (Table 2). On average, 2.27% of trials were deemed invalid because no valid response saccades were identified, and the two groups did not differ on the percentage of invalid trials. These findings suggest that basic saccade kinematics and response accuracy were similar between the two groups. Children with ASD and TD children both had reasonable understanding of and appropriate compliance with task instructions (see Figure 3 for group-averaged and individual psychometric functions and distributions of distance from saccade landing site to post-saccadic target location).

**Table 2.** Saccade metrics and task performance.

	TD (*n* = 35)	ASD (*n* = 30)		
	Mean ± SD	Mean ± SD	*t*(63)	*p* Value
*d'*	2.17 ± 0.52	2.15 ± 0.45	0.16	.87
PNL	0.21 ± 0.58	0.06 ± 0.38	1.26	.21
Mean saccade amplitude	8.87 ± 0.78	9.06 ± 0.95	–0.89	.38
Mean reaction time to first saccade	294.58 ± 76.64	298.37 ± 80.16	–0.20	.85
Mean variability in saccade end point	1.07 ± 0.35	1.08 ± 0.37	–0.18	.86
Percentage of invalid trials	1.90% ± 1.95%	2.69% ± 3.01%	–1.24	.22

Notes: ASD, children with autism spectrum disorder; df: degrees of freedom; TD, typically developing children.

### Reliance on saccade landing site in blanking task

There was evidence for significant main effects of target displacement, *F*(1, 84) = 402.12, *p* < 0.001; group, *F*(1, 96) = 5.49, *p* = 0.02; post-saccadic direction, *F*(1, 498) = 36.69, *p* < 0.001; and post-saccadic distance, *F*(1, 1283) = 27.85, *p* < 0.001 on perceptual judgments (see Supplementary Table S2). Holding other predictors constant across groups, for each 1° increase in target displacement, the odds of participants making a forward response increased by 3.81, consistent with our expectation. To examine the group effect, we computed simple slopes for ASD and TD groups separately. Results indicated that when target displacement was 0°, TD participants were significantly more likely to make a backward response, odds of forward response: 0.49, *t*(125) = −3.64, *p* < 0.001; whereas the ASD group had no response bias, that is, the odds of a forward response did not differ significantly from 1, *t*(75) = −0.43, *p* = 0.67. That is, TD children had a bias to report the target as jumping backwards, whereas children with ASD did not. To examine the post-saccadic direction effect, we computed simple slopes for post-saccadic locations that fell forward and backward of the landing sites separately. Results indicated that participants were significantly more likely to make a backward response when the post-saccadic target fell backward to the saccade landing site, odds of forward response: 0.27, *t*(467) = −4.69, *p* < 0.001, whereas participants had no response bias when the post-saccadic target fell forward to the saccade landing site, *t*(67) = −0.77, *p* = 0.45. Furthermore, holding other predictors constant, for each 1° increase in post-saccadic distance, the odds of participants making a forward response *decreased* by 0.30. In other words, when the post-saccadic target appeared farther from the saccade landing site, participants were less likely to make a forward response. These main effects are best interpreted in the context of several interaction effects.

There were four statistically significant two-way interaction effects: group × post-saccadic direction, *F*(1, 498) = 4.72, *p* = 0.03; target displacement × post-saccadic direction, *F*(1, 92) = 14.27, *p* < 0.001; target displacement × post-saccadic distance, *F*(1, 14645) = 33.00, *p* < 0.001; and post-saccadic direction × post-saccadic distance, *F*(1, 2008) = 14.95, *p* < 0.001. First, we explored the group × post-saccadic direction interaction. The effect of group was only statistically significant when the post-saccadic target jumped backward relative to saccade landing site, *t*(272) = 7.06, *p* = 0.008; on these backward trials, TD children had a significant backward response bias, odds of forward response, 0.27, *t*(467) = −4.69, *p* < 0.001, whereas children with ASD did not, *t*(67) = 1.08, *p* = 0.28. On forward appearing trials, there was no group difference in response bias, *t*(67) = 1.73, *p* = 0.19.

Next, we probed the target displacement × post-saccadic direction interaction. The effect of target displacement was statistically significant for both forward appearing, *F*(1, 77) = 500.23, *p* < 0.001, and backward appearing, *F*(1, 196) = 187.28, *p* < 0.001, trials, but with each 1° increase in target displacement, the odds of participants making a forward response was higher on forward trials than on backward trials (5.23 vs. 2.71). In other words, sensitivity to the target displacement was greater when the post-saccadic target appeared forward rather than backward of the saccade landing site.

Next, we explored the post-saccadic distance × post-saccadic direction interaction. Here, the effect of post-saccadic distance was statistically significant for both forward appearing, *F*(1, 71) = 7.25, *p* = 0.009, and backward appearing trials, *F*(1, 6142) = 22.58, *p* < 0.001, but with each 1° increase in post-saccadic distance away from the central fixation, the odds of participants making a forward response *decreased* more on backward trials than on forward trials (0.46 vs. 0.08). One potential interpretation here is that when the post-saccadic target appears far away from saccade landing sites, there is more spatial uncertainty owing to the post-saccadic target being far away from the fovea. Here, the higher the spatial uncertainty of the post-saccadic target, the less likely participants were to make a forward judgment.

To explore the target displacement × post-saccadic distance interaction, we calculated estimates based on small (1 SD below the mean) and large (1 SD above the mean) values of distance from the post-saccadic target. Results indicate that there was a significant target displacement effect for both small, *F*(1, 93) = 510.50, *p* < 0.001, and large, *F*(1, 192) = 187.70, *p* < 0.001, conditions, but with each 1° increase in target displacement, the odds of participants making a forward response was much higher when the distance between the saccade landing site and post-saccadic target was small than when it was large (5.69 vs. 2.46). In other words, participants were more sensitive to the actual target displacement when the post-saccadic locations appeared closer to the saccade landing site than when they appeared further away. This finding is consistent with a potential interpretation that, when there is more spatial uncertainty, participants are more likely to rely on prior assumptions about the target not moving (based on previous life experience that objects rarely move during the short duration of a saccade), thus rendering them less sensitive to actual target displacement.

There was a significant target displacement × post-saccadic direction × post-saccadic distance three-way interaction, *F*(1, 14645) = 11.56, *p* < 0.001. Estimates based on small and large distances from the post-saccadic target revealed that the target displacement × post-saccadic direction interaction was only statistically significant when the distance between saccade landing site and post-saccadic target was large, *F*(1, 451) = 18.35, *p* < 0.001. The simple slopes analyses revealed that only when the post-saccadic distance was large, was the effect of target displacement larger on forward appearing trials, *t*(83) = 15.53, *p* < 0.001, than on backward appearing trials, *t*(1266) = 3.03, *p* = 0.003: with each 1° increase in target displacement, the odds of participants making a forward response was much higher on forward trials than on backward trials (4.70 vs. 1.10). This interaction indicates an attenuated sensitivity to target displacement on backward as compared with forward trials, but only under conditions of high spatial uncertainty. Moreover, participants were significantly more likely to make a backward response on backward appearing trials, odds of forward response, 0.11, *t*(3445) = −4.58, *p* < 0.001, across all target displacements, indicating a reliance on saccade landing site to make perceptual judgments, but only when the post-saccadic distance was large (i.e., under conditions of high spatial uncertainty). The odds of a forward response was not significantly different from 1 on forward appearing trials, *t*(89) = −1.39, *p* = 0.17, whereas when the post-saccadic distance was small, there was no difference in the effect of target displacement between forward and backward appearing trials, nor was the overall likelihood of forward judgments across all target displacements significantly different from 1, *F*(1, 134) = 0.39, *p* = 0.54.

**Figure 4.**
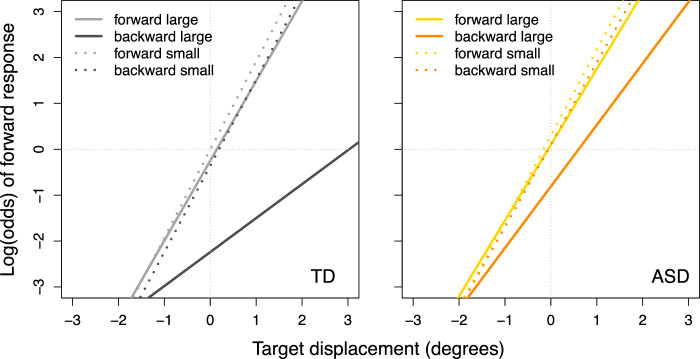
Group × Post-saccadic direction × Post-saccadic distance × Target displacement interaction effect. The units on the vertical axis represents the likelihood of making a forward response. Lines were plotted by computing a four-intercept model for each diagnostic group and each post-saccadic direction and then calculating estimates based on small (1 SD below the mean) and large (1 SD above the mean) post-saccadic distance. ASD, children with autism spectrum disorder; TD, typically developing children.

Finally, there was a significant group × target displacement × post-saccadic direction × post-saccadic distance four-way interaction, *F*(1, 14645) = 4.42, *p* = 0.04 (Figure 4). Results indicate that the target displacement × post-saccadic direction × post-saccadic distance interaction was statistically significant in the TD group, *F*(1, 14645) = 12.71, *p* < 0.001, but not in the ASD group, *F*(1, 14645) = 1.04, *p* = 0.31. We calculated estimates based on small and large values of post-saccadic distance and additionally computed simple slopes for post-saccadic target locations that appeared forward and backward to the saccade landing sites separately. Only in the TD group and only when the post-saccadic distance was large was the effect of target displacement larger on forward appearing trials than on backward appearing trials. This finding in TD children only suggests an attenuated sensitivity to target displacement on backward as compared with forward trials under conditions of high spatial uncertainty, *F*(1, 625) = 17.73, *p* < 0.001; when the post-saccadic distance was small, there was no difference in the effect of target displacement between forward and backward appearing trials in the TD group, *F*(1, 134) = 0.08, *p* = 0.77.

A simple summary of our interpretation of this complex pattern of data is the following: when the post-saccadic target appeared backward and far away from the saccade landing site (resulting in high spatial uncertainty), we made two (potentially related) observations that were especially prominent in the TD group. First, TD participants were less sensitive to target displacement, as compared with when the post-saccadic target appeared close to (low spatial uncertainty) and forward of the saccade landing site. One interpretation of this result is that there is greater influence of a prior that target displacement was 0 under conditions of high spatial uncertainty. We also found that TD participants were more likely to report the target as moving backward when the post-saccadic target fell backward of the saccade landing site, suggesting the use of saccade landing site as a proxy of the presaccadic target. These findings may suggest two potential strategies used by participants when spatial uncertainty of the target is high: (1) reliance on a prior that the target does not move and (2) use of landing site as a proxy of the presaccadic target location. Alternatively, use of the saccade landing site as a proxy for presaccadic target location may necessarily mean that they are relying less on target displacement, thus leading to a decreased sensitivity of displacement judgments to actual target displacement. Notably, and contrary to our hypothesis, sensitivity to target displacement in children with ASD was *less* reliant on the location of the post-saccadic target relative to saccade landing site, compared with TD children.

### Moderation effect of repetitive behaviors

We examined whether task performance varied as a function of restricted and repetitive behaviors as measured by the repetitive behaviors domain score from ADI-R in participants with ASD only. Full results are presented in Supplementary Table S3. Here we only present the significant main and interaction effects involving repetitive behaviors. We identified two significant two-way interactions, repetitive behaviors × post-saccadic direction, *F*(1, 116) = 7.67, *p* = 0.007; repetitive behaviors × post-saccadic distance, *F*(1, 4050) = 4.05, *p* = 0.04, two three-way interactions, repetitive behaviors × post-saccadic distance × target displacement, *F*(1, 5573) = 7.28, *p* = 0.007; repetitive behaviors × post-saccadic distance × post-saccadic direction, *F*(1, 171) = 4.33, *p* = 0.04), and one four-way interaction (repetitive behaviors × target displacement × post-saccadic distance × post-saccadic direction, *F*(1, 3265) = 15.03, *p* < 0.001 (see Supplementary Table S3). To unpack the nature of these interaction effects, we calculated estimates based on low (1 SD below the mean) and high (1 SD above the mean) ADI-R scorers. Results indicate that all five interactions were driven by the group with more severe repetitive behaviors. For children with ASD with more severe repetitive behaviors, there was a significant effect of post-saccadic direction, *F*(1, 95) = 18.39, *p* < 0.001, an effect of post-saccadic distance, *F*(1, 3040) = 12.58, *p* < 0.001, an interaction effect of post-saccadic distance × target displacement, *F*(1, 6687) = 17.66, *p* < 0.001, an interaction of post-saccadic distance × post-saccadic direction, *F*(1, 149) = 8.48, *p* = 0.004, and an interaction of target displacement × post-saccadic distance × post-saccadic direction, *F*(1, 5488) = 12.92, *p* < 0.001. None of these simple effects were statistically significant for children with ASD with less severe repetitive behaviors, post-saccadic direction, *F*(1, 114) = 0.13, *p* = 0.72; post-saccadic distance, *F*(1, 4453) = 0.46, *p* = 0.50; post-saccadic distance × target displacement, *F*(1, 6687) = 0.06, *p* = 0.81; post-saccadic distance × post-saccadic direction, *F*(1, 175) = 0.00, *p* = 0.95; and target displacement × post-saccadic distance × post-saccadic direction, *F*(1, 6687) = 3.28, *p* = 0.07).

For participants with ASD with more severe repetitive behaviors, we probed the post-saccadic direction effect by computing simple slopes for post-saccadic target locations that appeared forward and backward to the saccade landing sites separately. Our results indicate that, when the post-saccadic target appeared forward to the saccade landing site, participants with ASD with more severe repetitive behaviors were significantly more likely to make a forward response, odds of forward response, 1.75, *t*(35) = 2.62, *p* = 0.01, suggesting a reliance on saccade landing site to make perceptual judgments. In contrast, the odds of making a forward response on backward trials did not differ significantly from 1, *t*(79) = −1.46, *p* = 0.15. Next, we explored the post-saccadic distance effect for participants with more severe repetitive behaviors. Results indicated that, with each 1° increase in post-saccadic distance, the odds of participants with ASD with high repetitive behaviors making a forward response *decreased* by 0.24, *t*(3040) = −3.55, *p* < 0.001.

To explore the post-saccadic distance × target displacement interaction for participants with ASD with more severe repetitive behaviors, we computed estimates based on small (1 SD below the mean) and large (1 SD above the mean) values of distance from the saccade landing site. Results indicated that participants with ASD with high repetitive behaviors were more sensitive to the actual target displacement when the post-saccadic locations appeared closer to the saccade landing site, *F*(1, 52) = 168.86, *p* < 0.001, than when they appeared further away, *F*(1, 54) = 80.06, *p* < 0.001. Next, we explored the post-saccadic distance × post-saccadic direction interactions. Results revealed that in participants with ASD with high repetitive behaviors, the effect of post-saccadic distance was only statistically significant when post-saccadic target appeared backward to the saccade landing site, *F*(1, 1257) = −3.52, *p* < 0.001, with each 1° increase in post-saccadic distance away from the central fixation, the odds of participants making a forward response *decreased* by 0.41.

**Figure 5.**
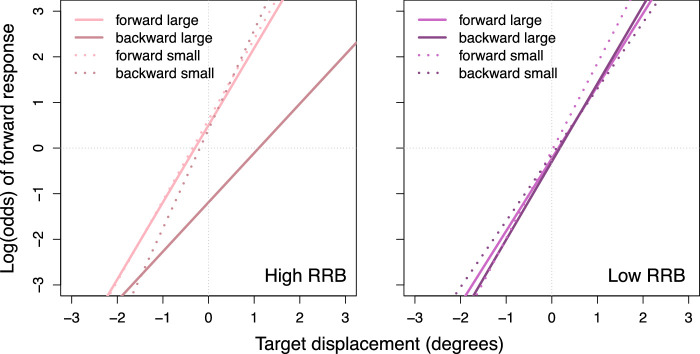
Repetitive behaviors × Target displacement × Post-saccadic direction × Post-saccadic distance interaction effect. The units on the vertical axis represents the likelihood of making a forward response. Lines were plotted by computing a two-intercept model for each direction (forward vs. backward) and then calculating estimates based on small (1 SD below the mean) and large (1 SD above the mean) post-saccadic distance and low (1 SD below the mean) and high (1 SD above the mean) repetitive behaviors. RRB, restricted and repetitive behaviors as measured by the repetitive behaviors domain score from the ADI-R.

To break down the target displacement × post-saccadic distance × post-saccadic direction interaction for participants with more severe repetitive behaviors, we calculated estimates based on small and large values of distance from the saccade landing site and additionally computed simple slopes for post-saccadic target locations that appeared forward and backward to the saccade landing sites separately (Figure 5). These results recapitulate the three-way interaction observed across all participants, but they were specific to a certain group: sensitivity to target displacement was greater when the post-saccadic target appeared close to (rather than far from) the saccade landing site (i.e., when spatial uncertainty was lower), but only in participants with ASD with more severe repetitive behaviors and only when the post-saccadic target appeared backward to the saccade landing site, *F*(1, 184) = 23.40, *p* < 0.001. Moreover, across all target displacements, participants with ASD with more severe repetitive behaviors were more likely to report the target as moving backwards relative to the presaccadic target when the post-saccadic target appeared backward and far away from their saccade landing site, odds of forward response, 0.30, *t*(403) = −2.76, *p* = 0.006. In other words, participants with ASD with more severe repetitive behaviors appear to have particular difficulties with perceptual judgments when the post-saccadic target appears backward to their saccade landing site and the spatial uncertainty of the target is high. In such cases, they were less sensitive to the actual target displacement and tended to rely heavily on saccade landing site and, potentially, on a prior that target displacement is zero.

We did not identify any statistically significant main effect of or interactions involving the RBS-R total score (see Supplementary Table S4).

### Moderation effect of sensory hyporesponsiveness

Finally, we examined whether task performance varied as a function of sensory responsiveness as measured by the hyporesponsiveness subscale total score from the SEQ. We did not identify any statistically significant main effect of or interactions involving the SEQ score (see Supplementary Table S5).

## Discussion

In the current study, we examined oculomotor CD signaling in children with ASD and TD children using a multilevel modeling approach to analyze performance during the blanking task. We found a group difference in the degree to which participants’ perceptual judgments were affected around the time of a saccade. Contrary to our hypothesis, however, children with ASD were *less* reliant on the location of the post-saccadic target relative to saccade landing site, compared with TD children, and more reliant on the actual target displacement. In line with our hypotheses, we also found that the severity of repetitive behaviors, which include motor symptoms, within the ASD group moderated participants’ task performance. Specifically, participants with ASD with more severe restricted and repetitive behaviors were less sensitive to the actual target displacement and more reliant on saccade landing site when the post-saccadic target appeared far away from the saccade landing site, likely resulting in a high spatial uncertainty. Finally, although task performance in all children looked very similar to what has been previously reported in adult clinical (Rösler et al., 2015) and nonclinical (Bansal, Jayet Bray, Peterson, & Joiner, 2015; Collins et al., 2009) samples, we did observe a complex interaction between target displacement and saccade landing site on perceptual judgments across the combined sample, enabled by a more powerful statistical approach than those used in previous studies. In this discussion, we interpret performance in this combined childhood sample in the context of previous studies, discuss the clinical findings, and consider potential limitations.

### Interaction between target displacement and saccade landing site

First, and most important, children as young as 8 years of age were able to perform this task and the results were remarkably similar to those in adult observers (see also Stewart, Hübner, & Schütz, 2020). We found a strong effect of target displacement on perceptual judgement, indicating that, when the target jumped forward, there was a high likelihood of reporting a forward jump. In addition, a multilevel modeling approach permitted us to test more complex interaction effects. This approach revealed a more nuanced influence of saccade landing site on perceptual judgements in this task than has been reported previously (Bansal et al., 2015; Collins et al., 2009; Rösler et al., 2015) and suggested that, under certain specific conditions, participants rely on information other than a correctly remapped location of the presaccadic target. Specifically, two observations emerged under conditions of higher spatial uncertainty and only when the post-saccadic target appeared backward to the saccade landing site. Compared with other conditions (i.e., when spatial uncertainty was low and/or the post-saccadic target appeared forward of the saccade landing site), participants were less sensitive to target displacement and more likely to report the target as moving backward on these trials. These findings may suggest two potential strategies used by participants when spatial uncertainty of the target is high: (1) reliance on a precise prior that the target does not move (and thus resulting in reduced sensitivity to target displacement) and (2) the use of landing site as a proxy of the presaccadic target location (and thus resulting in a bias of backward responses). Alternatively, decreased sensitivity to target displacement may be an inevitable consequence of using saccade landing site as a proxy for presaccadic target location, and thus may not reflect a different strategy for perceptual judgement (i.e., a reliance on a precise prior of 0 displacement). Notably, this observed effect seems to be present across both ASD and controls, but more prominent in the TD children.

An interaction between saccade landing site and target displacement has been observed in preclinical models. Joiner and colleagues (2013) found that non-human primates showed a dependence of perceptual judgements on saccade landing site in the blanking task, but only when target displacements were smaller—that is, when the perceptual decision was more difficult. Although the current results are in line with these findings in showing that a dependence on saccade landing site on perceptual judgements is moderated by the spatial configurations of presaccadic and post-saccadic targets, they are not entirely consistent. The discrepant results may be explained by factors related to age, species, and analytic strategy.

Why, under conditions of equally high spatial uncertainty, would findings of reduced sensitivity to target displacement and greater tendency to use saccade landing site as a proxy for the presaccadic target location be circumscribed to trials when the post-saccadic target appeared backward, but not forward, to where participants’ eyes landed? This finding could potentially be explained by the fact that our saccades are usually hypometric. In daily experience, individuals rarely encounter situations where their saccades overshoot a target by a large degree. They, therefore, have less experience in making perceptual judgements under these conditions than when their eyes fall short of a saccade target. It is possible that, in these rare conditions, participants waver between two opposite interpretations of the situation, either relying on the prior that objects usually do not move during a saccade (cf. the assumption of stability; Deubel et al., 1996) and assuming that the large distance between the saccade landing site and post-saccadic target location is due to intrinsic noise in their visuomotor system (e.g., errors in saccade planning or execution), or treating the large distance as evidence that the target did jump, and using their saccade landing site as a proxy of the presaccadic target. The current data support both interpretations. Recall that, when the post-saccadic target appeared far backward to where participants’ eyes landed, their sensitivity to target displacement was significantly attenuated. This finding is consistent with a greater reliance on a prior that the target does not move. At the same time, participants also exhibited a significant backward response bias on these trials across all target displacements. This finding is consistent with the interpretation of a greater reliance on the saccade landing site to localize the post-saccadic target. There is at least some evidence showing that children make less precise saccades than adults do, and therefore may have stronger expectations of greater intrinsic noise in the visuomotor system than adults (Stewart et al., 2020). However, more studies directly manipulating spatial uncertainty using experiments and/or computational modeling (e.g., Atsma, Maij, Koppen, Irwin, & Medendorp, 2016; Niemeier, Crawford, & Tweed, 2003) are needed to tease apart the relative contributions of a strong prior of target not moving versus a strong expectation of greater intrinsic noise.

### Effects of diagnostic group and repetitive behaviors

The degree to which diagnostic group moderated the aforementioned effects was in the opposite direction to what we predicted. When spatial uncertainty was high, TD children were less sensitive to the actual target displacement and more reliant on saccade landing site than participants with ASD were. One interpretation, contrary to our expectations, is that children with ASD were *more* effective than TD children at using CD signals to inform their perceptual judgments. In thinking about alternate explanations, however, we consider that CD is not the only source of eye position signals. Proprioceptive signals from the extraocular muscles (Masselink & Lappe, 2021; Sun & Goldberg, 2016; Ziesche & Hamker, 2011) and other external visual cues (e.g., boundary of the computer screen) can also provide such information. Thus, another potential explanation for our pattern of results is that participants with ASD were more effective in exploiting these alternate sources of eye position information, especially in situations where alternate sources may be the most useful (i.e., when spatial uncertainty is high). In fact, there is evidence suggesting intact (Fuentes, Mostofsky, & Bastian, 2011) or even superior proprioception (Paton, Hohwy, & Enticott, 2012) in individuals with ASD. Moreover, individuals with ASD selectively rely more on proprioceptive feedback (Izawa et al., 2012) over visual feedback (Sharer, Mostofsky, Pascual-Leone, & Oberman, 2016) in motor learning. Therefore, it is possible that, with more precise proprioceptive feedback, participants with ASD were better at ruling out the possibility that large trans-saccadic target jumps were due to intrinsic visuomotor noise and relied more on proprioceptive signals instead when making visual judgments.

Now, although our group effect was in a direction that was contrary to what we expected, with the reliance on saccade landing site being *less* prominent in children with ASD, these findings were nevertheless moderated by the severity of repetitive behaviors in the predicted direction. That is, participants with ASD with more severe repetitive behaviors seemed to have particular difficulties with perceptual judgments when the post-saccadic target appeared backward to their saccade landing site and the spatial uncertainty of the target was high. In such cases, those with more severe repetitive behavior symptoms were much less sensitive to the actual target displacement and tended to rely heavily on saccade landing site and, potentially, on a precise prior that target displacement is zero (i.e., that the target did not move). Because healthy participants rely on intact CD signals for predictive remapping of the presaccadic target location when making perceptual judgments immediately after a saccade (Collins et al., 2009), compromised CD signaling could lead to imprecise predictive remapping, and hence reduced sensitivity to target displacement (Thakkar, Diwadkar, & Rolfs, 2017; Thakkar & Rolfs, 2019), as observed here. One explanation for our findings then, is that CD signaling is compromised specifically in individuals with ASD and high levels of repetitive behavior symptoms (but less so in those with low levels of repetitive behavior symptoms), causing them to rely on their saccade landing site as a proxy of the presaccadic target location to make perceptual judgements. Alternatively, and as elaborated upon elsewhere in this article, children with more severe restricted and repetitive behaviors may have been less able to exploit alternate sources of information about eye gaze position (i.e., proprioception or external visual cues). In other words, an intact representation of the body (in this case, eye gaze) relative to the environment, subserved by CD signaling, proprioception, or use of external cues, may be a protective factor against severe restricted and repetitive behaviors in individuals with ASD.

### Interpretations in the context of Bayesian accounts of perception

These data may also be interpreted in the context of Bayesian accounts of perception, and trans-saccadic integration, in particular. The Bayesian model of perception proposes that perception is inherently inferential, given the inevitable noise and uncertainty in sensory input (Clark, 2013; Ma, 2012). Therefore, our brain maintains a model of the world with stored statistical regularities—priors—and combines them with the incoming sensory data to generate the most likely percepts. A prior can be quite vague (e.g., maybe there is a frog in that pond) or very precise (e.g., I am 100% sure there is a frog in that pond). A more precise prior has more influence on the eventual percept. Niemeier and colleagues (2003) proposed that trans-saccadic integration is likewise a Bayesian inference process: the post-saccadic percept (i.e., the perceived location of the post-saccadic target) is a posterior inference based on the combination of a prior (i.e., the probability of the target jumping) and the sensorimotor data (i.e., retinal image and CD signal). According to their model, one can obtain a veridical post-saccadic percept in the blanking task (here the actual target displacement is the ground truth) when they have highly reliable sensorimotor data (i.e., an accurate and precise CD signal and retinal image), and/or when they have a less precise prior of objects not moving during a saccade (that, therefore, more readily accommodates the possibility of the target jumping during the saccade). Therefore, high sensitivity to target displacement in the blanking task would suggest intact CD signaling, precise representation of target location, adjusted precision of prior in the context of the task, or a combination of these factors. On the contrary, decreased sensitivity to target displacement would suggest impaired CD signaling, noisy retinal image, unadjusted precision of the prior that static visual stimuli do not move during a saccade, or a combination of these factors.

Recall that we found an increased sensitivity to target displacement in children with ASD compared with TD children when the spatial uncertainty of the target was high. This group difference was driven by those children with ASD with less severe restricted and repetitive behaviors. We have considered that these findings may be driven by symptom-related differences in the integrity or use of CD signals (or alternate sources of eye position information). We now consider the possibility that the observed effects can be explained by group and individual differences in adjusting the precision of the prior that the target does not move.

The ability to flexibly adjust the precision of priors depending on the context is crucial to the balance between perception and action (Seth, 2015). Action can be beneficial to an inferential perception process, because sampling the environment can decrease the uncertainty, increase the reliability of incoming sensory data, and thereby increase the likelihood of obtaining a more veridical posterior inference. This strategy of engaging in more actions relies on decreasing the precision of prior temporarily, so that alternative hypotheses about the environment can be entertained. On the contrary, when the prior is highly precise, there is no need for more exploration, because the posterior inference will be heavily influenced by the prior. In the case of the blanking task, participants would be expected to start with a highly precise prior that the saccade target would not move owing to their past experience of stationary visual objects rarely changing location during a saccade. However, given the explicit experimental instruction (i.e., judging the direction of target displacement, implicating frequent target jumps) and the actual experimental experience, it is advantageous to lower the precision of this prior temporarily (for the duration of the task) to include a higher probability of target jumping (Atsma et al., 2016). Decreased sensitivity to target displacement in participants with ASD with more, as compared with less, severe restricted and repetitive behaviors, specifically when spatial uncertainty of the target is high indicates that, precisely when the environment is the most uncertain, they maintain an inflexibly precise prior of a less volatile external world. This interpretation is consistent with a Bayesian theory of autism, which proposes that a key ASD mechanism is an abnormality in context-sensitive precision modulation, which is influenced by the expected volatility of the world (Palmer, Lawson, & Hohwy, 2017). Such abnormality could potentially explain the symptoms of unusual fixation on specific objects and repetitive behaviors, including motor mannerisms and insistence on sameness, both as attempts to decrease volatility in the cause of sensory input and thereby in the uncertainty of the brain's model of the world (Palmer et al., 2017). Similarly, a recent study found atypical predictive visuomotor learning in children and adolescents with ASD that is consistent with a rigidly precise prior (Park, Schauder, Kwon, Bennetto, & Tadin, 2021). Because individuals with ASD with less severe restricted and repetitive behaviors actually showed enhanced sensitivity, compared with TD children, to target displacement under conditions of higher uncertainty, it may be that the ability to flexibly adjust the precision of priors serves a protective function that staves off more severe symptom presentation.

### Future directions and limitations

Several limitations need to be considered when interpreting the findings from the current study. First, we found that task performance was related to restricted and repetitive behaviors as measured by the ADI-R repetitive behaviors domain score, but not the RBS-R total score. This apparent discrepancy could potentially be explained by the fact that the ADI-R was rated by trained psychologists, but the RBS-R is a self-report questionnaire filled out by caregivers of the participants with ASD. The psychologists had a wider reference frame for rating symptoms and thus the ADI-R may be less biased by parental judgement and more accurately reflect the severity of ASD symptoms, thereby yielding a clearer association with task performance. Second, there is no sensory processing measure normed for ASD individuals in the age range of current study sample. Consequently, we used the closest to ideal measure (Ausderau et al., 2014). It is possible that a relationship between task performance and sensory hyporesponsiveness could be identified if there is a more appropriate measure that could better capture the nuance of sensory processing disturbances in this age range. A third consideration is the specific neurodevelopmental stage that we sampled (i.e., from older childhood to adolescence). There is recent evidence showing that the saccadic suppression of displacement effect (i.e., the inability to perceive small stimulus displacement during a saccade) is stronger in TD children (7–12 years old) than it is in healthy adults, with or without the stimulus blanking (Stewart et al., 2020), indicating that the mechanisms supporting trans-saccadic perception continue to develop throughout childhood. Future studies examining samples at later neurodevelopmental stages and/or longitudinally are needed to reach a more comprehensive understanding of the development of oculomotor CD signaling in ASD and its putative association with motor and sensory symptoms. Last, the ASD sample in this study was relatively high functioning and did not represent the full range of the functional spectrum in ASD. In fact, one would not expect children with severe motor and/or sensory symptoms to be able to complete the rather tedious psychophysics paradigm in its current form successfully. Therefore, this selective subset of participants may have contributed to a restricted range of sampling, rendering findings of the current study less generalizable to a more symptomatic sample. Nevertheless, the current findings would be bolstered by replication studies to establish a more solid link between altered CD signaling and motor and sensory processing deficits in ASD.

## Conclusions

We found that, compared with TD participants, children with ASD were more sensitive to target displacement and less reliant on saccade landing site when spatial uncertainty of the post-saccadic target was high. These results were driven by the ASD participants with less severe restricted and repetitive behaviors, who may have had better compensatory strategies such as enhanced use of proprioceptive cues or more flexible priors. This finding suggests a potential mechanistic link between CD signaling and core motor symptoms in ASD and may point toward more targeted interventions for repetitive behaviors via enhancing the link between action and perception.

## Supplementary Material

Supplement 1
