# Supplementary material for: Oculomotor corollary discharge signaling is related to repetitive behavior in children with autism spectrum disorder
Source: J Vis. 2021 Aug 5;21(8):9. doi: 10.1167/jov.21.8.9 (PMC8354038; doi:10.1167/jov.21.8.9)
Supplement: Supplement 1 [file jovi-21-8-9_s001.pdf]

Supplementary Material

Corrective Saccade Analysis

We examined saccades to the post-saccadic target and whether they differed between groups or influenced task performance. We defined a corrective saccade as the first saccade following the initial task-related saccade (i.e., the saccade starting from the fixation point towards the pre-saccadic target), with an onset later than the onset of post-saccadic target, and which brought the eye closer to the post-saccadic target location. On average, participants made a corrective saccade on 33.05% of the trials with a valid task response, and typically developing (TD) participants and participants with autism spectrum disorder (ASD) did not differ in either the incidence of these corrective saccades or their amplitude (**Table S1**). We also investigated the threshold for executing a corrective saccade. Consistent with prior reports (Ostendorf, Liebermann, & Ploner, 2010), there was a close relationship between post-saccadic distance and corrective saccade amplitude in all participants (**Figure S1**). We used two methods to calculate each individual's threshold for corrective saccade initiation. First, we selected all trials where a corrective saccade was initiated and identified the minimum value of the post-saccadic distance (i.e., the absolute value of the distance between saccade landing site and the post-saccadic target location) as the threshold. Second, we computed the percentage of corrective saccades as a function of post-saccadic distance by fitting a logistic function (with an asymptote parameter) to each participant's data (**Figure S2**). Threshold was defined as the post-saccadic distance value at the inflection point of the logistic curve. The two groups did not differ in threshold for corrective saccade initiation using either method (**Table S1**). Combined, these findings suggest that basic saccade kinematics of corrective saccades and the distance between gaze location and the post-saccadic target that triggers a corrective saccade were similar between the two groups.

We next examined whether executing a corrective saccade influenced task performance and sensitivity to target displacement by including corrective saccade, corrective saccade × target displacement, and corrective saccade × target displacement × group into the first analysis model. Corrective saccade was coded as 0 = no corrective saccade, 1 = corrective saccade identified. There was no effect of corrective saccade ($F(1, 14645) = 0.00$, $p = .94$), corrective saccade × target displacement ($F(1, 14645) = 0.35$, $p = .56$), and corrective saccade × target displacement × group ($F(1, 14645) = 1.61$, $p = .20$). All other significant effects reported in the main Results remained significant. Moreover, the previously marginally significant target

displacement × post-saccadic distance × group interaction effect became statistically significant in this model (F(1, 14645) = 4.71, p = .03). To explore this interaction, we calculated simple slopes for ASD and TD groups and estimates based on small (1 SD below the mean) and large (1 SD above the mean) values of distance from the post-saccadic target. Results indicated that post-saccadic distance moderated sensitivity to target displacement in both groups (TD: F(1, 14645) = 26.78, p < .001; ASD: F(1, 14645) = 6.84, p = .009) such that participants' sensitivity was higher when the post-saccadic distance was small than when the distance was large. However, the moderation effect was stronger in the TD group (i.e., their sensitivity to target displacement was much lower when the post-saccadic distance was large). This interaction effect is consistent with the target displacement × post-saccadic distance × post-saccadic direction × group interaction effect reported in the Results section of the main text.

Moderation effect of IQ

Given that previous literature identified a relationship between IQ and restricted and repetitive behaviors (RRBs; Bishop, Richler, & Lord, 2006), we examined this relationship and potential moderation effect of IQ on task performance in ASD participants. In the ASD group, we did not find a correlation between IQ and RRBs (ADI-R: r_s = -0.02, p = .90; RBS-R: r_s = -0.21, p = .28). After adding IQ to the ADI-R model, there was no significant change to model fit, and IQ was not a significant predictor of task performance (F(1, 28) = 0.15, p = .70). Adding the IQ x ADI-R interaction did improve the model fit (x^2(2)=38.67, p < .001), but the interaction effect was not statistically significant either (F(1, 29) = 1.37, p = .25). Similarly, adding IQ and the IQ x RBS-R interaction term improved the model fit of the RBS-R model (IQ: x^2(1)=18.52, p < .001; IQ & IQ x RBS-R: x^2(2)=20.88, p < .001) but neither effect was statistically significant (IQ: F(1, 26) = 0.19, p = .66; IQ x RBS-R: F(1, 26) = 0.12, p = .74). Therefore, it seems that IQ did not moderate task performance in this ASD sample, nor could it account for the relationship between task performance and RRBs.

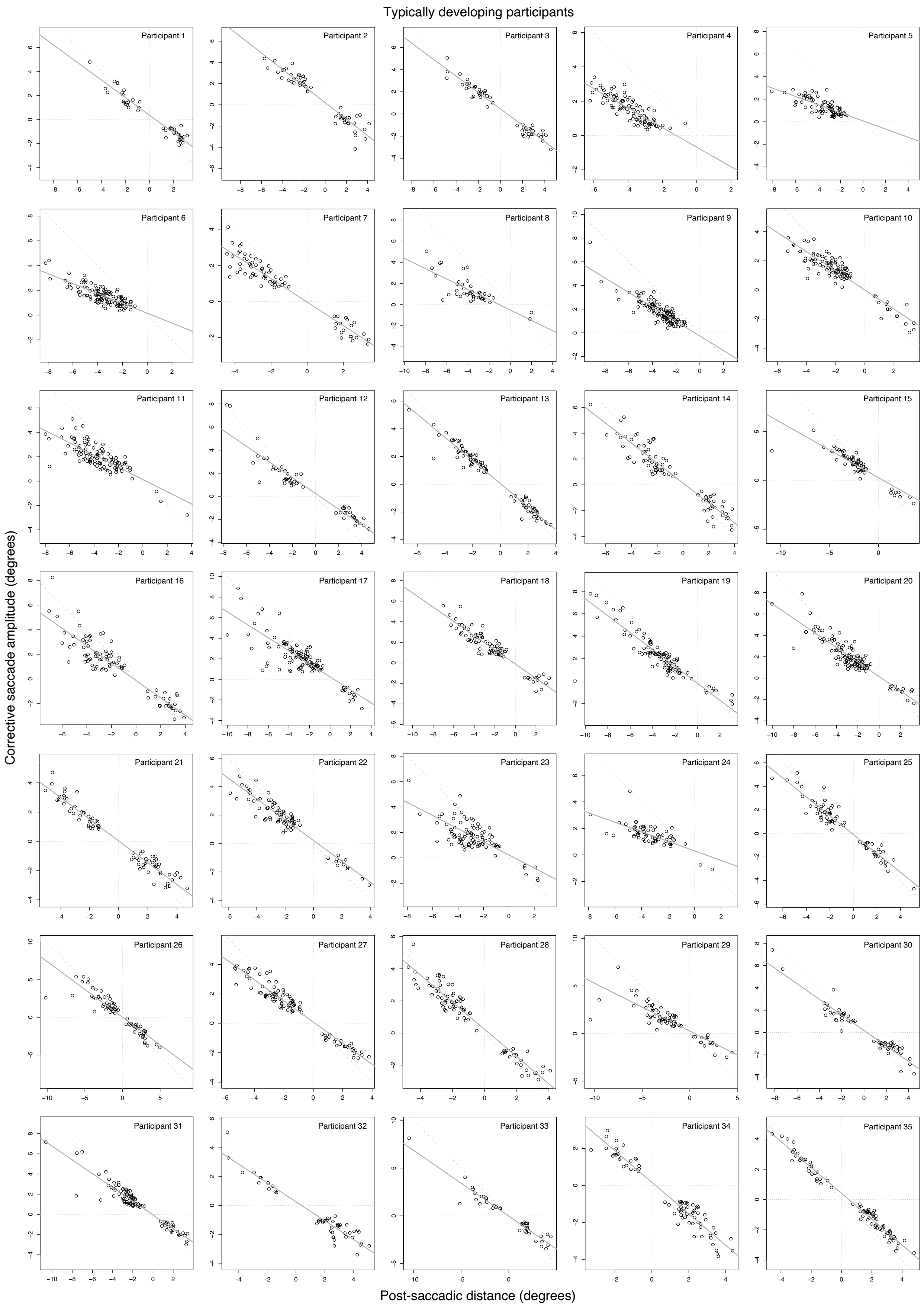
Typically developing participants
Participant 1
Participant 2
Participant 3
Participant 4
Participant 5
Participant 6
Participant 7
Participant 8
Participant 9
Participant 10
Participant 11
Participant 12
Participant 13
Participant 14
Participant 15
Participant 16
Participant 17
Participant 18
Participant 19
Participant 20
Participant 21
Participant 22
Participant 23
Participant 24
Participant 25
Participant 26
Participant 27
Participant 28
Participant 29
Participant 30
Participant 31
Participant 32
Participant 33
Participant 34
Participant 35
Corrective saccade amplitude (degrees)
Post-saccadic distance (degrees)

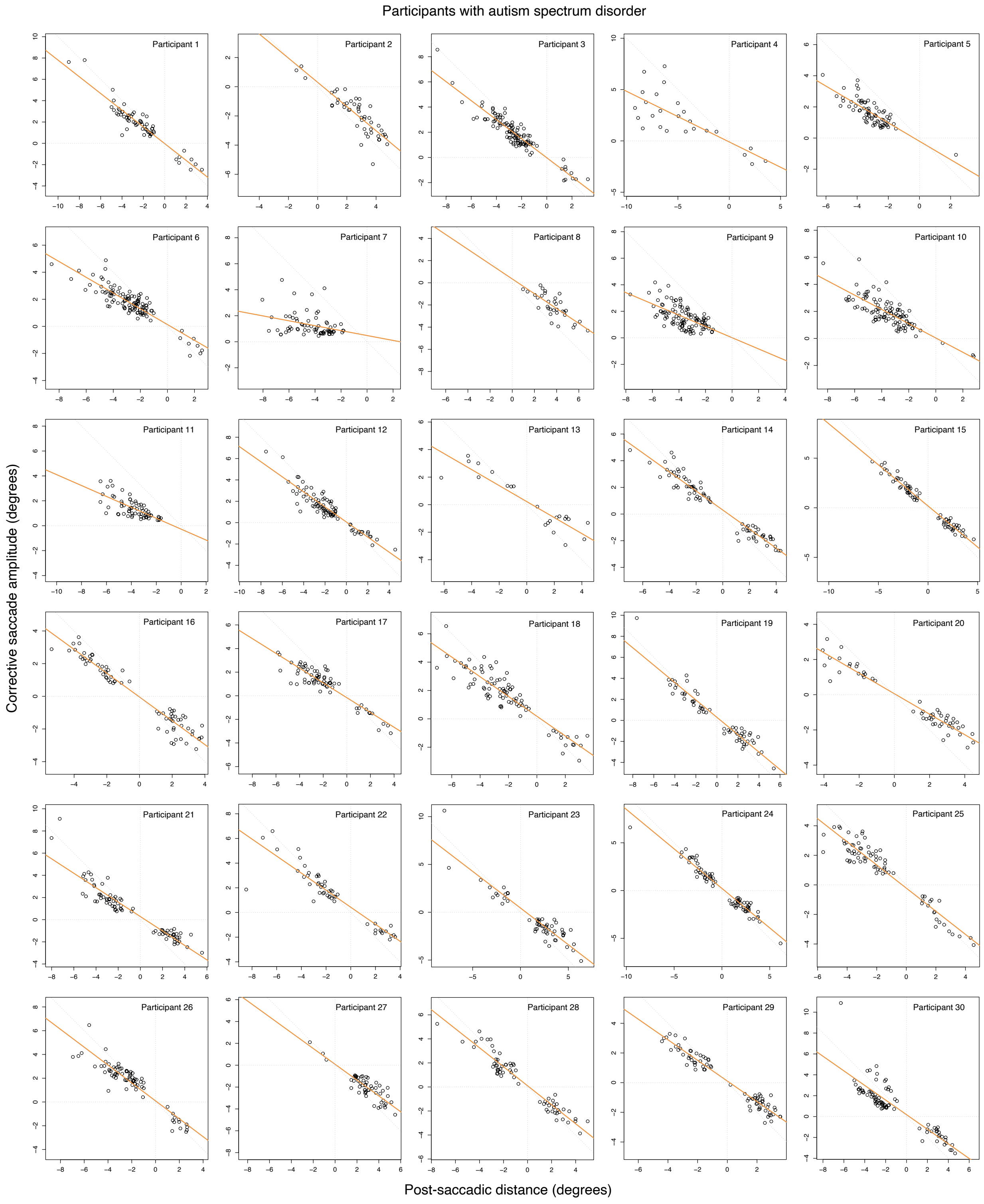

Figure S1. Individual scatterplots of corrective saccade amplitude (negative = moving away from initial fixation, positive = moving towards initial fixation) against post-saccadic distance (negative = post-saccadic target appeared backward to the initial saccade landing site, positive = post-saccadic target appeared forward to the landing site). The grey and orange lines are the

simple regression lines predicting corrective saccade amplitude from post-saccadic distance for TD and ASD participants, respectively. The thin dotted line is the identity line.

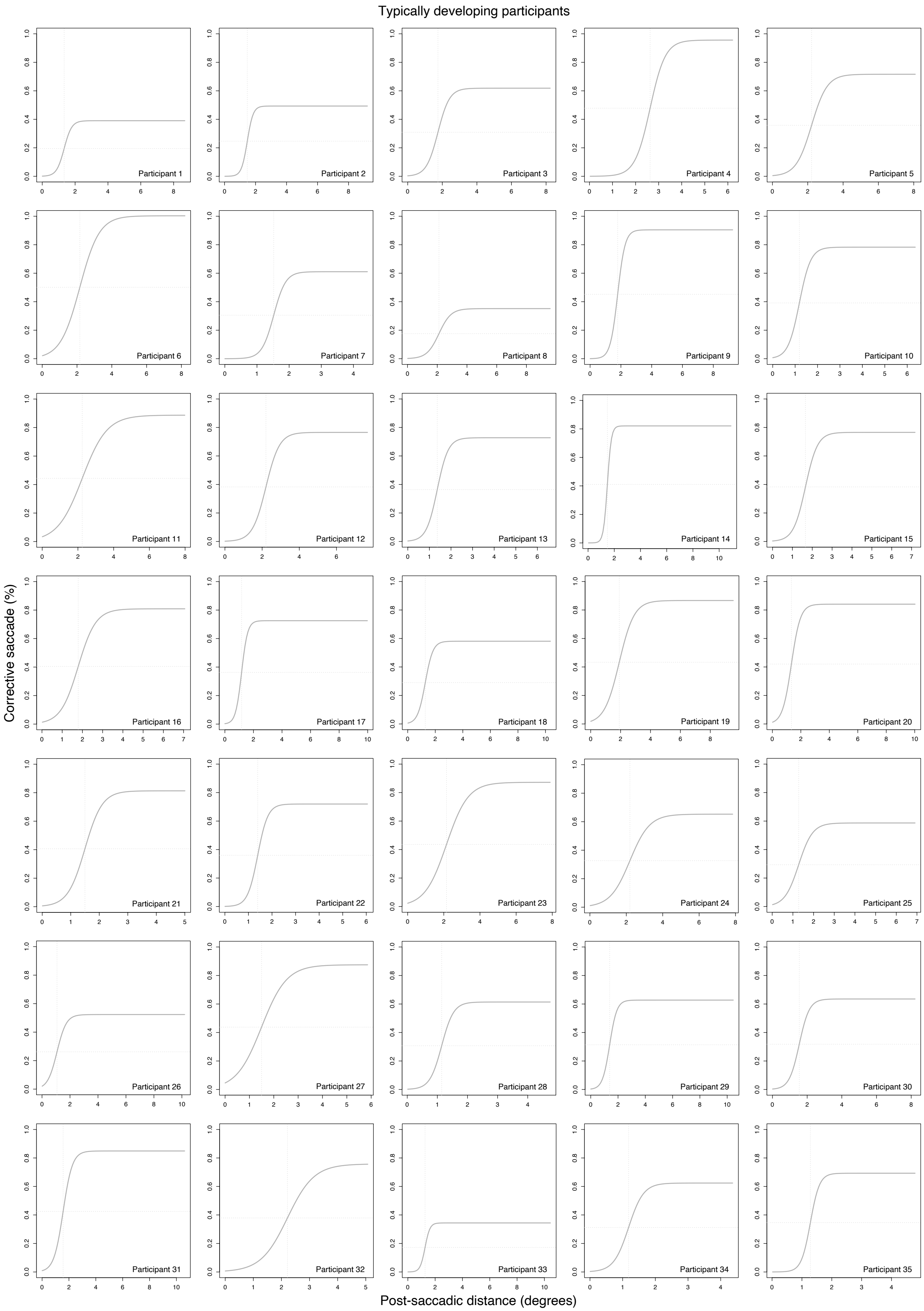
Typically developing participants
Participant 1
Participant 2
Participant 3
Participant 4
Participant 5
Participant 6
Participant 7
Participant 8
Participant 9
Participant 10
Participant 11
Participant 12
Participant 13
Participant 14
Participant 15
Participant 16
Participant 17
Participant 18
Participant 19
Participant 20
Participant 21
Participant 22
Participant 23
Participant 24
Participant 25
Participant 26
Participant 27
Participant 28
Participant 29
Participant 30
Participant 31
Participant 32
Participant 33
Participant 34
Participant 35
Corrective saccade (%)
Post-saccadic distance (degrees)

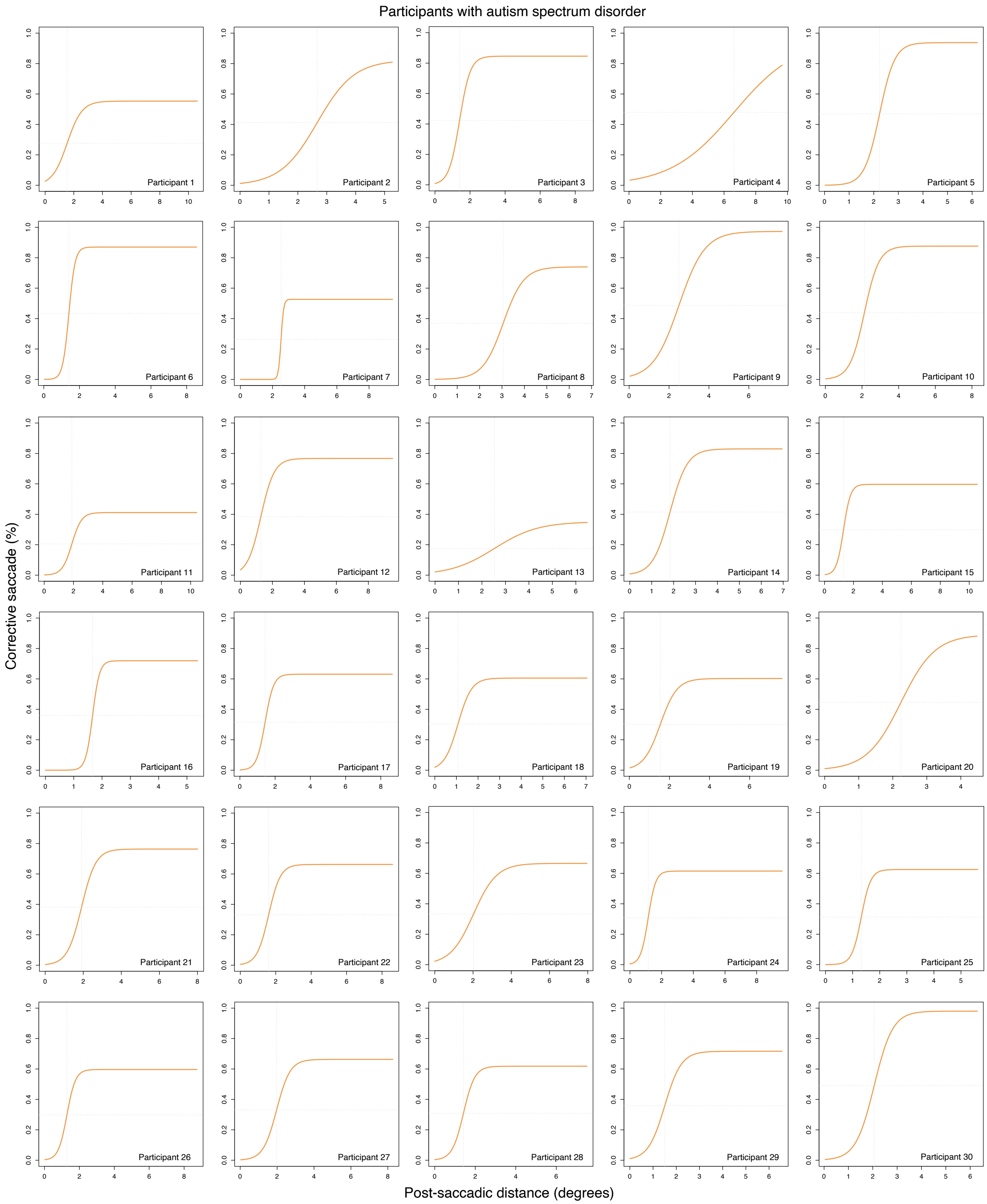

Figure S2. Individual logistic fits of percentage of corrective saccades as a function of post-saccadic distance (the absolute value of the distance between saccade landing site and the post-saccadic target location).

Table S1. Corrective saccade metrics.

	TD ($N = 35$)	ASD ($N = 30$)		
	Mean ± SD	Mean ± SD	$t(63)$	p
Percentage of valid trials with a corrective saccade	34.79% ± 9.18%	31.03% ± 9.17%	-1.65	.11
Mean corrective saccade amplitude	1.89 ± 0.25	1.96 ± 0.30	1.03	.31
Mean post-saccadic distance threshold (minimum value)	0.84 ± 0.28	0.90 ± 0.35	0.84	.40
Mean post-saccadic distance threshold (inflection point)	1.64 ± 0.41	1.97 ± 1.01	1.79	.08

Table S2. Logistic multilevel regression coefficients estimating effects of group, saccade direction, target displacement, post-saccadic direction, and post-saccadic distance on perceptual judgment on the blanking task

Variable	*b*	Odds	*F(df)*	σ^2	*Wald Z*
Fixed Effects					
Intercept	-0.72	0.49			
Group			5.49* (96)		
Saccade Direction	0.08	1.08	0.02 (326)		
Target Displacement	1.57	4.81	402.12*** (84)		
Post-saccadic Direction	-0.59	0.55	36.69*** (498)		
Post-saccadic Distance	-0.35	0.70	27.85*** (1283)		
Group × Saccade Direction			0.67 (326)		
Group × Target Displacement			0.32 (84)		
Group × Post-saccadic Direction			4.72* (498)		
Group × Post-saccadic Distance			2.75 (1283)		
Saccade Direction × Target Displacement	0.08	1.08	0.01 (187)		
Saccade Direction × Post-saccadic Direction	-0.06	0.94	0.78 (768)		
Saccade Direction × Post-saccadic Distance	-0.03	0.97	0.14 (892)		
Target Displacement × Post-saccadic Direction	-0.26	0.77	14.27*** (92)		
Target Displacement × Post-saccadic Distance	-0.22	0.80	33.00*** (14645)		
Post-saccadic Direction × Post-saccadic Distance	-0.27	0.76	14.95*** (2008)		
Group × Saccade Direction × Target Displacement			3.00 (187)		
Group × Saccade Direction × Post-saccadic Direction			0.00 (768)		

Group × Saccade Direction × Post-saccadic Distance			0.07 (892)		
Group × Target Displacement × Post-saccadic Direction			2.94 (92)		
Group × Target Displacement × Post-saccadic Distance			3.53 (14645)		
Group × Post-saccadic Direction × Post-saccadic Distance			2.31 (2008)		
Post-saccadic Direction × Saccade Direction × Target Displacement	0.004	1.00	0.25 (4570)		
Post-saccadic Distance × Saccade Direction × Target Displacement	0.08	1.08	1.23 (14645)		
Post-saccadic Direction × Saccade Direction × Post-saccadic Distance	-0.04	0.96	0.59 (10334)		
Post-saccadic Distance × Post-saccadic Direction × Target Displacement	-0.16	0.85	11.56*** (14645)		
Group × Saccade Direction × Target Displacement × Post-saccadic Direction			0.36 (4570)		
Group × Saccade Direction × Target Displacement × Post-saccadic Distance			2.42 (14645)		
Group × Saccade Direction × Post-saccadic Distance × Post-saccadic Direction			0.00 (10334)		
Group × Target Displacement × Post-saccadic Distance × Post-saccadic Direction			4.42* (14645)		
Saccade Direction × Target Displacement × Post-saccadic Distance × Post-saccadic Direction	0.06	1.06	0.23 (14645)		
Group × Saccade Direction × Target Displacement × Post-saccadic Direction × Post-saccadic Distance			2.20 (14645)		
Random Effects					
Intercept				0.89***	5.22
Saccade Direction				0.11***	3.83
Target Displacement				0.28***	4.36

Post-saccadic Direction	0.01	0.57
Post-saccadic Distance	0.004	0.77
Saccade Direction × Target Displacement	0.03*	1.94
Saccade Direction × Post-saccadic Direction	0.02	1.31
Saccade Direction × Post-saccadic Distance	0.01*	1.65
Target Displacement × Post-saccadic Direction	0.01	0.91
Post-saccadic Direction × Post-saccadic Distance	0.0004	0.08

*$p < .05$, **$p < .01$, *** $p < .001$.

Table S3. Logistic multilevel regression coefficients estimating effects of past and current repetitive behaviors (measured by ADI-R), target displacement, post-saccadic direction, and post-saccadic distance on perceptual judgment on the blanking task in ASD participants

Variable	*b*	Odds	*F(df)*	σ^2	*Wald Z*
Fixed Effects					
Intercept	-0.05	0.95			
ADI-R	0.08	1.08	0.81 (39)		
Target Displacement	1.67	5.31	294.27*** (36)		
Post-saccadic Direction	-0.26	0.77	10.40** (94)		
Post-saccadic Distance	-0.17	0.84	8.99** (3135)		
ADI-R × Target Displacement	0.003	1.00	0.00 (34)		
ADI-R × Post-saccadic Direction	-0.13	0.88	7.67** (116)		
ADI-R × Post-saccadic Distance	-0.07	0.93	4.05* (4050)		
Target Displacement × Post-saccadic Direction	-0.07	0.93	1.45 (3673)		
Target Displacement × Post-saccadic Distance	-0.10	0.90	7.94** (6687)		
Post-saccadic Direction × Post-saccadic Distance	-0.12	0.89	4.15* (151)		
ADI-R × Target Displacement × Post-saccadic Direction	0.01	1.01	0.08 (3659)		
ADI-R × Target Displacement × Post-saccadic Distance	-0.05	0.95	7.28** (5573)		
ADI-R × Post-saccadic Direction × Post-saccadic Distance	-0.07	0.93	4.33* (171)		
Post-saccadic Distance × Post-saccadic Direction × Target Displacement	-0.04	0.96	0.94 (6687)		
ADI-R × Target Displacement × Post-saccadic Distance × Post-saccadic Direction	-0.08	0.92	15.03*** (3265)		
Random Effects					
Intercept				0.56***	3.48

Target Displacement	0.18**	2.67
Saccade Direction	0.12**	2.73
Post-saccadic Direction	0.01	0.61
Saccade Direction × Target Displacement	0.06*	2.15
Saccade Direction × Post-saccadic Distance	0.01	1.29
Post-saccadic Direction × Post-saccadic Distance	0.01	0.91

*$p < .05$, **$p < .01$, *** $p < .001$.
ADI-R: Autism Diagnostic Interview – Revised.

Table S4. Logistic multilevel regression coefficients estimating effects of current repetitive behaviors (measured by RBS-R), target displacement, post-saccadic direction, and post-saccadic distance on perceptual judgment on the blanking task in ASD participants

Variable	*b*	Odds	*F(df)*	σ^2	*Wald Z*
Fixed Effects					
Intercept	-0.06	0.94			
RBS-R	-0.009	0.99	0.70 (37)		
Target Displacement	1.68	5.37	216.07*** (33)		
Post-saccadic Direction	-0.24	0.79	7.52** (78)		
Post-saccadic Distance	-0.14	0.87	5.56* (1754)		
RBS-R × Target Displacement	-0.009	0.99	1.58 (31)		
RBS-R × Post-saccadic Direction	0.00	1.00	0.00 (95)		
RBS-R × Post-saccadic Distance	-0.002	1.00	0.15 (1705)		
Target Displacement × Post-saccadic Direction	-0.08	0.92	1.58 (3590)		
Target Displacement × Post-saccadic Distance	-0.08	0.92	4.56* (6246)		
Post-saccadic Direction × Post-saccadic Distance	-0.09	0.91	2.11 (137)		
RBS-R × Target Displacement × Post-saccadic Direction	-0.008	0.99	3.38 (3506)		
RBS-R × Target Displacement × Post-saccadic Distance	-0.002	1.00	0.77 (6246)		
RBS-R × Post-saccadic Direction × Post-saccadic Distance	-0.001	1.00	0.04 (137)		
Post-saccadic Distance × Post-saccadic Direction × Target Displacement	-0.01	0.99	0.13 (6246)		
RBS-R × Target Displacement × Post-saccadic Distance × Post-saccadic Direction	0.002	1.00	0.39 (6246)		
Random Effects					

Intercept	0.56***	3.31
Target Displacement	0.19**	2.59
Saccade Direction	0.10**	2.44
Post-saccadic Direction	0.03	0.97
Saccade Direction × Target Displacement	0.06*	2.08
Saccade Direction × Post-saccadic Distance	0.01	1.21
Post-saccadic Direction × Post-saccadic Distance	0.005	0.51

p* < .05, ***p* < .01, * *p* < .001.
RBS-R: Repetitive Behavior Scale-Revised.

Table S5. Logistic multilevel regression coefficients estimating effects of sensory hypo-responsiveness (measured by SEQ), target displacement, post-saccadic direction, and post-saccadic distance on perceptual judgment on the blanking task in ASD participants

Variable	*b*	Odds	*F(df)*	σ^2	*Wald Z*
Fixed Effects					
Intercept	-0.10	0.90			
SEQ	0.01	1.01	0.81 (39)		
Target Displacement	1.66	5.26	265.60*** (34)		
Post-saccadic Direction	-0.25	0.78	8.42** (92)		
Post-saccadic Distance	-0.16	0.85	7.38** (1705)		
SEQ × Target Displacement	0.004	1.00	0.15 (30)		
SEQ × Post-saccadic Direction	0.009	1.01	1.01 (98)		
SEQ × Post-saccadic Distance	-0.001	1.00	0.06 (1351)		
Target Displacement × Post-saccadic Direction	-0.08	0.92	1.62 (4252)		
Target Displacement × Post-saccadic Distance	-0.10	0.90	7.01** (6453)		
Post-saccadic Direction × Post-saccadic Distance	-0.10	0.90	2.92 (157)		
SEQ × Target Displacement × Post-saccadic Direction	-0.006	0.99	1.04 (3158)		
SEQ × Target Displacement × Post-saccadic Distance	-0.002	1.00	0.39 (6453)		
SEQ × Post-saccadic Direction × Post-saccadic Distance	-0.003	1.00	0.29 (157)		
Post-saccadic Distance × Post-saccadic Direction × Target Displacement	-0.01	0.99	0.14 (6453)		
SEQ × Target Displacement × Post-saccadic Distance × Post-saccadic Direction	0.003	1.00	0.51 (6453)		
Random Effects					

Intercept	0.53***	3.39
Target Displacement	0.19**	2.63
Saccade Direction	0.12**	2.71
Post-saccadic Direction	0.03	0.98
Saccade Direction × Target Displacement	0.06*	2.09
Saccade Direction × Post-saccadic Distance	0.01	1.20
Post-saccadic Direction × Post-saccadic Distance	0.002	0.25

*$p < .05$, **$p < .01$, *** $p < .001$.
SEQ: Sensory Experience Questionnaire.

References

Bishop, S. L., Richler, J., & Lord, C. (2006). Association Between Restricted and Repetitive Behaviors and Nonverbal IQ in Children with Autism Spectrum Disorders. *Child Neuropsychology*, *12*(4–5), 247–267. https://doi.org/10.1080/09297040600630288

Ostendorf, F., Liebermann, D., & Ploner, C. J. (2010). Human thalamus contributes to perceptual stability across eye movements. *Proceedings of the National Academy of Sciences*, *107*(3), 1229–1234. https://doi.org/10.1073/pnas.0910742107
